# Correlated electronic decay in expanding clusters triggered by intense XUV pulses from a Free-Electron-Laser

**DOI:** 10.1038/srep40736

**Published:** 2017-01-18

**Authors:** Tim Oelze, Bernd Schütte, Maria Müller, Jan P. Müller, Marek Wieland, Ulrike Frühling, Markus Drescher, Alaa Al-Shemmary, Torsten Golz, Nikola Stojanovic, Maria Krikunova

**Affiliations:** 1 Institut für Optik und Atomare Physik, Technische Universität Berlin, Strasse des 17. Juni 135, ER 1-1, 10623 Berlin, Germany; 2 Max-Born-Institut, Max-Born-Straße 2a, 12489 Berlin, Germany; 3 Department of Physics, Imperial College London, South Kensington Campus, SW7 2AZ London, United Kingdom; 4 Institut für Optik und Atomare Physik, Technische Universität Berlin, Hardenbergstr. 36, EW 3-1, 10623 Berlin, Germany; 5 Institut für Experimentalphysik, Universität Hamburg, Luruper Chaussee 149, 22761 Hamburg, Germany; 6 Center for Ultrafast Imaging, Luruper Chaussee 149, 22761 Hamburg, Germany; 7 Deutsches Elektronen-Synchrotron DESY, Notkestraße 85, 22607 Hamburg, Germany

## Abstract

Irradiation of nanoscale clusters and large molecules with intense laser pulses transforms them into highly-excited non- equilibrium states. The dynamics of intense laser-cluster interaction is encoded in electron kinetic energy spectra, which contain signatures of direct photoelectron emission as well as emission of thermalized nanoplasma electrons. In this work we report on a so far not observed spectrally narrow bound state signature in the electron kinetic energy spectra from mixed Xe core - Ar shell clusters ionized by intense extreme-ultraviolet (XUV) pulses from a free-electron-laser. This signature is attributed to the correlated electronic decay (CED) process, in which an excited atom relaxes and the excess energy is used to ionize the same or another excited atom or a nanoplasma electron. By applying the terahertz field streaking principle we demonstrate that CED-electrons are emitted at least a few picoseconds after the ionizing XUV pulse has ended. Following the recent finding of CED in clusters ionized by intense near-infrared laser pulses, our observation of CED in the XUV range suggests that this process is of general relevance for the relaxation dynamics in laser produced nanoplasmas.

Within femtoseconds, nanoscale objects exposed to intense ultrashort light pulses are transformed into highly-excited non-equilibrium states^1^,^2^,^3^. As a result, a dense spatially confined nanoplasma is created which relaxes and finally disintegrates into charged fragments on femtosecond to picosecond time-scales^2^,^4^,^5^. It is of fundamental interest to understand the dynamics of highly excited plasma states, which is also essential for a variety of practical applications including the control of sample integrity in diffractive imaging experiments^1^,^3^,^6^.

The excitation and ionization of atoms embedded into an environment opens a variety of additional deexcitation channels. Quasi-free energetic electrons excited within nanoscale samples and bulk solids thermalize through multielectron as well as lattice collisions^4^,^7^. Another deexcitation mechanism is related to an autoionization of excited atomic states. By this mechanism an excited atom relaxes and the excess energy is used to ionize the same or a neighboring atom, or it is transferred to a quasi-free electron in the environment. Interatomic (or intermolecular) Coulombic decay (ICD) is a well-known example of such an ultrafast and highly efficient autoionization processes in which at least two atoms are involved. ICD was predicted by Cederbaum *et al*.^8^ and since then has been subjected to extensive theoretical and experimental investigations (see e.g. refs 9, 10, 11, 12 for reviews). For instance, low-energy ICD-electrons produced upon ionization of water dimers^13^ and water clusters^14^ are considered as a source for radiation damage in biological matter. An example for a practical application is the characterization of heterogeneous structures and interfaces using spectral signatures attributed to ICD^15^,^16^. The high density of atomic excited states populated within a single cluster also results in collective autoionization processes in which several excited atoms are involved as predicted theoretically^17^ and observed experimentally at a free electron laser (FEL)^18^,^19^,^20^.

In most previous studies, precursors for ICD or other autoionizing channels are populated directly through resonant or nonresonant absorption of extreme ultraviolet (XUV) photons by individual atoms within a molecule, dimer, or larger cluster^9^,^10^,^11^,^12^. In the X-ray photon energy range a release of the inner-shell electron may cause a collective excitation of valence electrons (shake-up) or release of the outer valence electron (shake-off). Shake-up and shake-off excitations are shown to be very efficient in large biomolecules^21^. Another important mechanism by which excited atomic states can be populated very efficiently was, however, not discussed until recently. In clusters and large molecules exposed to intense laser fields, complex relaxation processes in the dense nanoplasma also lead to the formation of a large population of atoms and ions in a Rydberg states^22^,^23^,^24^. Recently, spectral signatures of these states were used to track electron-ion recombination dynamics of highly excited nanoplasma^22^,^23^. New experimental findings confirm that during the cluster expansion excited atomic states can relax by autoionization^25^,^26^. Alternatively, ICD or a process involving a quasi-free nanoplasma electron can take place^27^. In superfluid He nanodroplets^28^ autoionization might also be caused by collisions of excited atoms and is referred to as a Penning ionization^29^. To account for a broad family of electron correlation processes that may take place in clusters, a general term - correlated electronic decay (CED) - was introduced^27^. The CED term describes energy exchange between at least two Rydberg electrons which are located either within the same doubly excited atom (autoionization) or between electrons located in different atoms (ICD or Penning ionization). Additionally, CED may take place between a Rydberg and a quasi-free nanoplasma electron. In previous studies of CED^25^,^26^,^27^, nanoplasma was created upon ionization of clusters with near-infrared (NIR) pulses. Considering that electron-ion recombination in nanoplasma is a common process, which occurs irrespective of the ionization mechanisms^23^,^30^, it should also be possible to observe CED in disintegrating clusters ionized by intense XUV or X-ray FEL-pulses.

Here we report on the investigation of intense XUV laser-cluster interactions at a photon energy of 92 eV, i.e. far above the atomic ionization threshold. As a target system Xe-Ar core-shell clusters were studied in comparison with pristine Xe clusters and atoms. Characteristic spectral signatures are observed in the electron spectra of mixed Xe-Ar clusters, which are attributed to CED process involving Ar atoms. We use the terahertz (THz) field streaking principle^31^ to discern CED-electrons from electrons directly activated by the XUV pulse. In contrast to photo- and Auger electrons, CED-electrons are found not to be streaked by the THz field, demonstrating that the latter are emitted at least several picoseconds after the XUV pulse has terminated. Our results are of general relevance for FEL experiments that investigate nanoscale particles using intense XUV and X-ray light.

## Results

**Figure 1:**
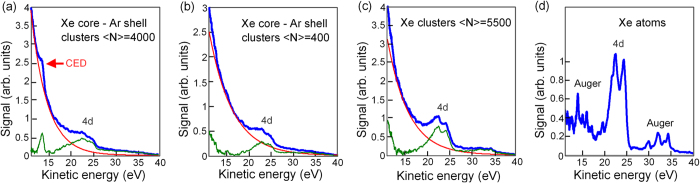
Electron kinetic energy spectra measured after ionization of clusters (**a**–**c**) and atoms (**d**) with intense FEL pulses. Each spectrum (blue solid line) is an average of about 300 single FEL shots. Contributions of 4d core-levels of Xe (**a**–**d**) as well as lines corresponding to the Auger decay in Xe (**d**) are visible. All cluster spectra (**a**–**c**) show a contribution from thermal electrons characterized by near to an exponential distribution. Red solid line shows an exponential function used to subtract the thermal electron contribution in Fig. 2. The corresponding difference spectra are shown in green. A peak structure at ≈13.7 eV is well pronounced above a thermal electron distribution in Xe core - Ar shell clusters with 〈*Ν*〉 = 4000 atoms (**a**). This signature is attributed to correlated electronic decay (CED).

Figure 1 shows electron kinetic energy spectra recorded from core-shell clusters with an average size of 〈*Ν*〉 = 4000 (a) and 〈*Ν*〉 = 400 (b) atoms in comparison to pristine Xe clusters (〈*Ν*〉 = 5500 atoms) (c) and isolated Xe atoms (d). The core-shell structures consist of a Xe core covered by 3–4 and 1–2 layers of Ar for larger and smaller clusters, respectively^32^,^33^,^34^. All spectra were measured after irradiation of the target with XUV pulses with a photon energy of 92 eV and an estimated average intensity in the interaction region of 2 × 10^15^ W/cm^2^. In the spectra from Xe atoms (Fig. 1(d)) contributions from 4d_3/2_ and 4d_5/2_ core-levels of Xe at 22 and 24 eV as well as lines corresponding to the NOO Auger decay in Xe^35^ are observed. We note that electron spectra below 10 eV are not shown because the transmission of our time-of-flight spectrometer drops down significantly in this spectral region.

The broadening of 4d-photolines in the cluster spectra with respect to the atomic case is mostly explained by inelastic collisions of escaping electrons with the environment as well as by a mixture of surface and bulk contributions^36^. In cluster spectra Auger lines are obscured by the thermal electron contribution. Due to the fast growing Coulomb potential only a small amount of electrons can escape from the cluster. Most of the activated electrons become trapped inside the cluster and form a nanoplasma. These electrons thermalize very quickly by exchanging their energy in multiple collisions^2^,^4^,^5^,^37^. In photoelectron spectra thermalized electrons appear as a smooth distribution, which is generally modeled by an exponential function^5^,^37^,^38^. In the spectra of larger core-shell clusters an additional peak structure at a kinetic energy of ~13.7 eV on a top of a thermalized electron distribution is clearly discernible (Fig. 1(a)). The observation of a clear bound-state signature, which is indicative for a transition with a very well defined initial and final state, in this kinetic energy range is surprising. It cannot be attributed to NOO Auger decay in Xe because these lines can hardly be resolved in spectra from pristine Xe clusters (Fig. 1(c)). Moreover, in highly excited systems any bound-state signature is usually strongly smeared by the space-charge or also by collective phenomena such as collective autoionization^20^.

In order to access the time-scale of the electron emission from atoms and clusters, we have performed a THz-field streaking measurement. The effect of the light-field-streaking can be understood as an additional momentum acquired by the free electron in the presence of a dressing electric field^39^,^40^. By changing the time-delay between the XUV pulse with respect to the streaking field and measuring electron kinetic energy spectra, a streaking spectrogram is obtained. For femtosecond XUV pulses available at the FEL in Hamburg (FLASH) the THz radiation generated in the dedicated THz-undulator^41^ is used for streaking^31^.

**Figure 2:**
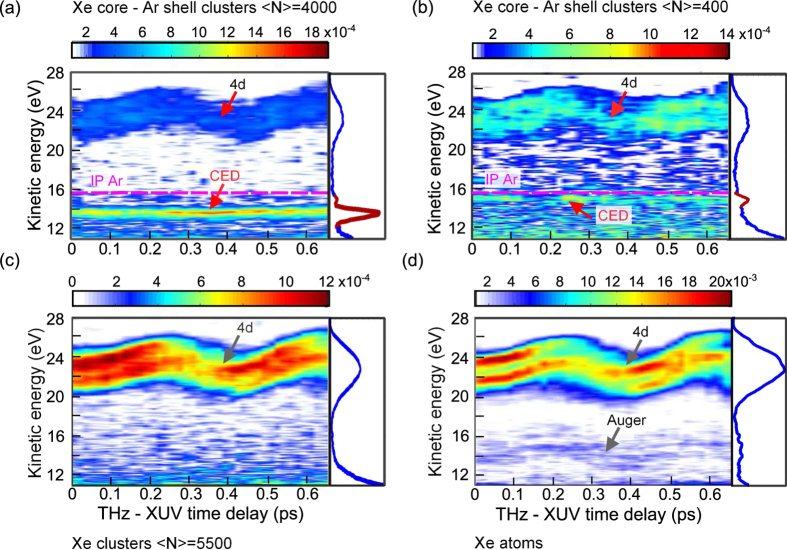
THz streaking spectrograms recorded in cluster (**a**–**c**) and atomic (**d**) targets. The panels on the right side of each streaking spectrogram show the corresponding electron spectra integrated over all THz-XUV time-delays. In the spectrograms recorded in clusters (**a**–**c**) only nonthermal contributions to the electron kinetic energy spectra are shown. The oscillatory behavior of the 4d-photolines in all spectrograms as well as of the Auger-region in (**d**) is due to a shift in the electron momentum induced by the THz field. In Xe core - Ar shell clusters the signatures at ~13.7 eV in (**a**) and at ~15 eV in (**b**) are close to the ionization potential (IP) of Argon atoms (15.8 eV), and, are assigned to the correlated electronic decay (CED). CED-signatures do not show an oscillatory behavior, demonstrating that these electrons are emitted on a time-scale of several picoseconds after excitation.

Figure 2(a–d) shows THz streaking spectrograms that were obtained following ionization of the same targets under the same experimental conditions as the THz field free electron spectra shown in Fig. 1(a–d). The smooth thermal contributions of electrons in the cluster spectra (Fig. 2(a–c)) were subtracted in order to increase the visibility of the bound-state signatures (description on subtraction procedure can be found in methods). The 4d-electrons in Fig. 2(a–d) as well as the Auger electrons in Fig. 2(d) show a characteristic oscillatory behavior due to the shift in electron momentum induced by the instantaneous THz field at the instance of electron emission^39^,^40^. Different widths of the 4d-photolines at the ascending and the descending slopes of the streaking traces are attributed to a negative frequency chirp of XUV pulses which is characteristic for FEL pulses^31^,^42^. Surprisingly, however, the emission line at ~13.7 eV (Fig. 2(a)) in larger Xe-Ar clusters does not show an oscillatory behaviour, and, thus, is insensitive to the THz streaking field. Also in the streaking spectrogram from smaller Xe-Ar clusters, a feature with a similar behaviour at slightly higher kinetic energy ~15 eV is visible in Fig. 2(b), which was not clearly resolvable in the THz field free spectra (Fig. 1(b)). We will address this point in the discussion section.

Both signatures become more pronounced in photoelectron spectra integrated over all THz-XUV time-delays (red highlighted in Fig. 2(a,b), right panels). While the 4d_3/2_ and 4d_5/2_ photolines as well as the Auger-lines are broadened and smeared in time-delay integrated spectra due to their oscillatory behaviour, the peaks at ~13.7 eV and ~15 eV remain narrow (Fig. 2, right panels). Note that the observed features are very close to the first ionization threshold of Ar atoms at 15.76 eV. The insensitivity of the features at ~13.7 eV and ~15 eV in Fig. 2(a) and (b) to the THz streaking field indicates that these electrons must have a different origin compared to 4d and Auger electrons, as they are emitted on a longer time-scale compared to the THz pulse duration. The THz pulse duration is in the order of several picoseconds^31^,^41^. At this time-scale electron-ion recombination in a nanoplasma takes place and the cluster has significantly expanded^6^,^23^,^24^,^43^,^44^,^45^.

**Figure 3:**
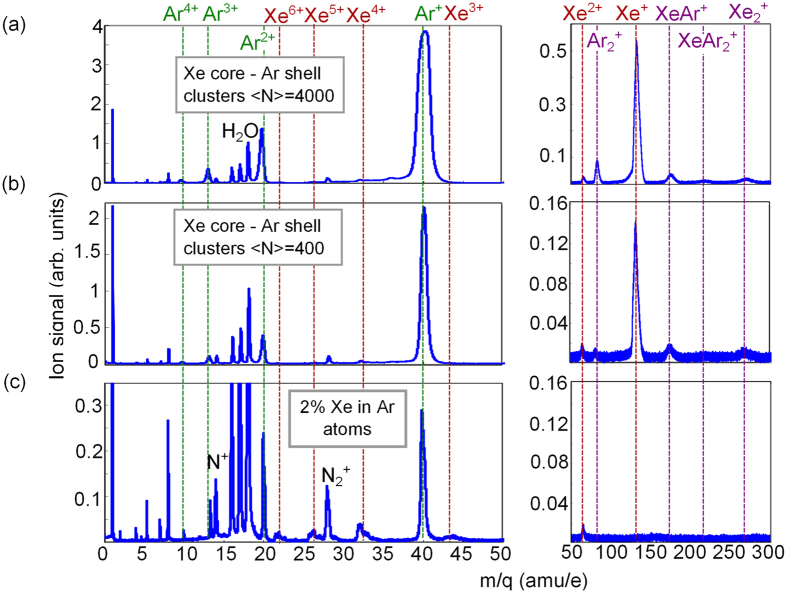
Ion spectra after ionization of Xe core - Ar shell clusters (**a**–**b**) and Xe - Ar gas mixture (**c**) with intense FEL pulses. Spectra are plotted on a mass-to-charge (m/q) scale. Left part shows spectra with small and right part with large m/q ratio, respectively. Each spectrum is an average over about 300 single FEL shots, and is normalized to the peak of H_2_O^+^ background signal at m/q = 18. Note that all spectra are dominated by the Ar^+^ ion contribution.

In the ion spectra in Fig. 3 one can observe that electron-ion recombination is highly efficient. Among Xe charge states the signal from Xe^+^ prevails (Fig. 3(a,b)), even though at a photon energy of 92 eV, i.e. within the 4d → *ε*f giant resonance, one-photon ionization of the 4d shell leads to Xe^2+^ and Xe^3+^ final states in atoms^46^. Note that almost no Xe^+^ ions are observed in atomic spectra (Fig. 3(c)). Instead, atomic spectra show substantial contributions of Xe 4+, 5+ and 6+ states, which are indicative for a sequential absorption of several photons at our irradiation conditions^47^. An additional indication for electron-ion recombination processes in clusters is the presence of singly charged Xe and Ar dimers as well as mixed oligomers in ion cluster spectra (Fig. 3(a,b), right panel). The Ar^+^ charge state dominates in Xe-Ar cluster spectra, similar to a previous work on Xe-Ar mixed clusters^33^. Even though the absorption cross-section of Xe in this photon energy range is much higher (24.7 Mb compared to 1.4 Mb for Ar^46^), the ratio of Xe to Ar atoms in clusters is about one to ten^32^,^33^,^34^. Therefore, the large fraction of Ar^+^ ions is indicative for a more efficient electron-ion recombination inside the Xe core as compared to the Ar shell^33^,^45^.

## Discussion

**Figure 4:**
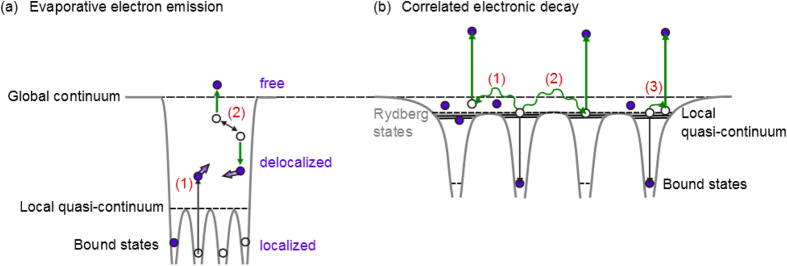
Electron emission processes from highly ionized clusters. (**a**) Evaporative electron emission. Upon photoabsorption electrons are excited from bound states into the local quasi-continuum (process 1), where they can exchange energy through collisions. In this way some of the electrons can overcome the trapping potential and leave the cluster via evaporative emission (process 2). The dynamics of delocalized electrons in the local quasi-continuum is modeled by considering electrons as classical particles^5^,^24^,^30^,^37^. The distribution of these electrons in photoelectron spectra is characterized by an exponential decay function^5^,^37^. (**b**) Correlated electronic decay (CED) involving two weakly bound electrons, where one electron relaxes from a Rydberg state to the atomic ground state. The energy can be transferred to an electron in a local quasi-continuum (process 1), to a Rydberg electron bound by a neighbouring (process 2) or by the same (process 3) atom. In all cases, one electron can escape from the cluster. The dynamics of CED is described by quantum mechanics^8^,^9^,^17^. If the cluster has expanded significantly, the kinetic energies of escaped CED-electrons can be close to an atomic ionization potential^27^.

Figure 4 schematically shows two fundamentally different electron emission processes taking place in highly ionized clusters after an ultrashort laser pulse has ended. In the nanoplasma, electrons move as quasi-free particles and can classically exchange energy through collisions (Fig. 4(a)). The exponential contribution to the electron kinetic energy spectra from clusters as observed in Fig. 1(a,c) is related to this evaporative electron emission. Evaporative electron emission in electron spectra was previously observed in experiments at FELs^20^,^37^ as well as at HHG sources^22^,^48^ and is accounted for by theoretical models^4^,^5^,^24^,^30^,^37^. However, electrons may also exchange energy via correlation, as sketched in Fig. 4(b). By this mechanism one electron relaxes from a highly excited to an atomic ground state and the excess energy is transferred either to a quasi-free nanoplasma electron (process 1 in Fig. 4(b)) or to a second excited electron in a Rydberg state bound by a neighbouring (process 2) or by the same (process 3) atom. The dynamics of the CED-process requires a quantum mechanical description^8^,^9^,^17^,^49^,^50^.

In numerous previous studies of clusters under intense XUV and (soft) X-ray radiation the electron photoemission by CED-mechanism has not been taken into account^2^,^4^,^5^,^30^,^37^. In the current study the new unexpected features at ~13.7 eV and ~15 eV kinetic energy in photoelectron spectra of Xe-Ar clusters (Fig. 2) are attributed to the CED process between Ar atoms, as shown in Fig. 4(b). The assignment is based on the following experimental findings. First, the spectral distribution of CED-electrons shows a very sharp peak structure which is indicative for transitions with a well-defined initial and final state. Second, the kinetic energy of observed electrons is close to the first ionization potential of Ar indicating that Ar atoms in highly excited states are involved in CED. CED-electrons are found to be insensitive to the THz streaking field and, thus, they are emitted with at least a few picoseconds delay with respect to the XUV ionization of a cluster.

It is not clear whether the observed dynamics is slow because of a slow population or slow decay rate of high-lying Rydberg states because in our study population and decay rates cannot be differentiated. It is probable that in contrast to most of previous studies of correlated electron dynamics^9^,^10^,^11^,^12^,^51^,^52^,^53^,^54^,^55^ the precursors for observed CED-channels are populated few picoseconds after the absorption of XUV-photons. On this time-scale the clusters are substantially expanded and resemble a strongly diluted plasma^24^,^43^. Indeed, classical molecular dynamics simulations of the temporal evolution of single-particle-energy spectra show that upon cluster expansion and nanoplasma cooling the electron distribution evolves into three energetically well separated contributions consisting of loosely bound electrons with the energy close to the continuum (Rydberg electrons and quasi-free electrons bound by the cluster potential) as well as electrons bound to neutral ground state atoms and singly ionized ions^24^,^27^. The loosely bound electrons are considered to be involved in CED^27^. A slow decay rate would be in contrast to other time-resolved measurements of electron correlation processes. For instance, Auger decay^51^,^52^ or ICD in dimers^53^,^55^ were shown to take place on femtosecond time-scales. However, in the case of ICD in dimer a virtual photon model predicts a substantial increase of the ICD lifetime for higher excited states^27^ in accordance with experimental observation^54^.

We note that CED-electrons emitted on femtosecond to few picosecond time-scale cannot be observed because their kinetic energy is strongly influenced by the space-charge of the cluster thus smearing out the characteristic bound-state signature. Notably, in larger clusters the CED-peak at ~13.7 eV is shifted towards lower kinetic energies with respect to the CED-peak at ~15 eV observed in smaller clusters. In smaller clusters the CED-peak is located closer to the first ionization threshold of Ar atoms at ~15.76 eV. This finding is in accordance with the expectation that the kinetic energy of CED-electrons might be influenced by the cluster environment. Indeed, larger clusters disintegrate more slowly^23^,^43^,^44^,^45^, and, thus, the longer influence of the charged cluster environment may result in CED-peak broadening and a shift towards lower electron kinetic energy. We note that in the current study the CED-signature in spectra of smaller clusters is not as pronounced as in larger clusters. We attribute this finding to the nanoplasma formation in the Ar outer shell. To observe a well pronounced signature of CED-electrons in kinetic energy spectra a substantial fraction of neutral atoms in highly excited states have to be produced. It was discussed in ref. 33 that for smaller clusters a nanoplasma is mainly formed in the Xe core, while for larger clusters the nanoplasma can extend into the Ar shell. Therefore, efficient Rydberg atom and ion formation is expected to take place in the Ar shell only for larger clusters making the CED process in smaller clusters less probable.

CED-electrons observed in the current study exhibit similar dynamics as found very recently in clusters irradiated with intense NIR-pulses^27^. In spite of very different ionization mechanisms taking place during laser-cluster interaction in XUV and NIR regime, the nanoplasma thermalization and cluster disintegration in both cases leads to the formation of a large fraction of atoms in excited states. Therefore, CED is, probably, a process commonly taking place in expanding clusters. In order to gain a deeper understanding of electron correlation processes in nanoscale particles interacting with intense laser pulses, a substantial extension of theoretical models is crucial. So far, the dynamics induced by intense laser-cluster interactions is modeled semiclassically^5^,^24^,^30^,^37^. Therein, atomic ionization processes are treated quantum mechanically via corresponding cross-sections, whereas the dynamics of the resulting electrons and ions is treated classically. Our results, however, imply that it is important to account for quantum phenomena such as CED. Here our measurements can serve as benchmark results of CED on long (picosecond) timescales, to which the results of model calculations can be compared to.

In summary, we have presented to the best of our knowledge the first demonstration of correlated electronic decay in expanding clusters triggered by intense XUV pulses from a FEL. CED is manifested by the observation of characteristic un-modulated peaks in THz-streaked electron kinetic energy spectra, which clearly discloses an electron emission delayed by at least few picoseconds. Our results indicate that CED is of general relevance for the recombination dynamics in highly ionized nanoscale particles. The observation of CED-process discussed in the current study is not restricted to experiments at FELs. With advances of state-of-the-art laser driven HHG (high harmonic generation) sources in combination with THz or NIR probe pulses such experiments become feasible in laboratory-scale environment^22^,^48^ as well as at emerging user facilities such as Extreme Light Infrastructure (ELI beamlines and ELI ALPS)^56^.

## Methods

The experiments were performed at the beamline BL3 of the FEL in Hamburg (FLASH), which provides intrinsically synchronized XUV and THz pulses^41^,^57^. The experimental geometry was similar to the one described in ref. 31. Briefly, at the experimental end-station XUV pulses of 92 eV photon energy (corresponding to 13.5 nm central wavelength) and THz-pulses of 2.5 THz central frequency (corresponding to 120 *μ*m wavelength) were collinearly overlapped. For balancing the XUV and THz beamline lengths the XUV pulses were delayed and back focused by a spherical molybdenum/silicon multilayer mirror with a focal length of 2.1 m to a spot size of about 10 *μ*m. The XUV pulse duration of 100 fs was estimated from a THz-streaking measurement in Ne atoms using the method described in ref. 31. With an average XUV pulse energy of 150 *μ*J the estimated irradiation intensity in the interaction region was around 2 × 10^15^ W/cm^2^.

THz pulses were focused with an off-axis parabolic mirror of 320 mm focal length to a spot size of 1.8 mm (FWHM). The maximal THz electric field strength of 8 MV/m and the corresponding intensity of 8.3 × 10^6^ W/cm^2^ was estimated from streaking spectrograms. To obtain streaking spectrograms photoelectron spectra were measured parallel to the THz polarization direction with a time-of-flight spectrometer. The relative time-delay between XUV and THz pulses was varied in 30 fs steps by the delay line available in the THz-branch^41^. At each XUV - THz time-delay photoelectron spectra were averaged over about 100 single FEL shots.

Electron kinetic energy spectra were measured with a home-built spectrometer of a similar design as used in ref. 31. It consists of several electrostatic lenses connected to the flight tube. According to the simulation with the SIMION software the spectrometer transmission within the 10 to 45 eV kinetic energy range is almost flat with a slight linear increase towards higher kinetic energies. A linear function derived from SIMION-simulation was used to correct the spectra in Fig. 1 (blue solid line).

A pulsed cluster beam was generated by adiabatic expansion of either pure Xe or a gas premix of 2% Xe in Ar through a 100 *μ*m conical nozzle (15° half-opening angle) at room temperature. The average cluster size was controlled by adjusting the stagnation pressure behind the nozzle and quantitatively estimated with the help of scaling laws^58^. To maintain the pressure within the experimental chamber at 5 × 10^−7^ mbar the pulsed cluster jet was double skimmed. A stagnation pressure of 6 bar was used to produce pristine Xe clusters comprising of 〈*Ν*〉 = 5500 atoms. Due to differences in binding energies and melting points of Xe and Ar atoms, a core of Xe atoms nucleates and becomes surrounded by Ar atoms at the surface of the clusters. As a result well-defined core-shell structures can be produced^32^. Smaller and larger Xe core - Ar shell clusters of 〈*Ν*〉 = 400 and 4000 atoms were prepared by expanding the gas premix at 7 and 17 bars, respectively. According to previous studies^32^,^33^,^34^ the estimated Xe enrichment factor was around 10. This resulted in the core-shell structures roughly consisting of 80 and 800 Xe atoms in the core with 1–2 and 3–4 Ar layers on top of it, respectively.

Cluster spectra in Fig. 1 are dominated by a contribution from thermal electrons characterized by near to an exponential distribution. To increase the visibility of 4d and CED-signatures in color plots of Fig. 2 we have tried to subtract the thermal electron contribution. For this we have used a single exponential function which best fits to the spectrum in 12 to 18 eV range as well as fits to the base-line in 40 to 45 eV range (red solid line in Fig. 1). Difference spectra are shown in green (Fig. 1). This procedure has been applied to each spectrum in streaking spectrograms (Fig. 2). The described procedure allows only partial thermal background subtraction. A tail in Fig. 1(b) and (c) at low kinetic energies indicates that the electron emission profile has a more complex structure than assumed here.

## Additional Information

**How to cite this article**: Oelze, T. *et al*. Correlated electronic decay in expanding clusters triggered by intense XUV pulses from a Free-Electron-Laser. *Sci. Rep.* **7**, 40736; doi: 10.1038/srep40736 (2017).

**Publisher's note:** Springer Nature remains neutral with regard to jurisdictional claims in published maps and institutional affiliations.
